# Supplementary material for: Double burden of malnutrition in Nepal: A trend analysis of protein-energy malnutrition and High Body Mass Index using the data from Global Burden of Disease 2010–2019
Source: PLoS One. 2022 Sep 29;17(9):e0273485. doi: 10.1371/journal.pone.0273485 (PMC9521909; doi:10.1371/journal.pone.0273485)
Supplement: Attachment — Submitted filename: Response to Reviewers.docx [file pone.0273485.s002.docx]

**Response to Reviewers**

We thank the editor and two reviewers for their comments on the manuscript. Please find below our responses to each point raised by academic editor and reviewers. We are also submitting an updated version of our manuscript. We hope that we satisfyingly addressed them and that the manuscript will be now suited for publication.

Sincerely,

On behalf of all authors,

Priza Pradhananga

**Academic editor:**

1. Please ensure that your manuscript meets PLOS ONE's style requirements, including those for file naming. The PLOS ONE style templates can be found at

<https://journals.plos.org/plosone/s/file?id=wjVg/PLOSOne_formatting_sample_main_body.pdf> and <https://journals.plos.org/plosone/s/file?id=ba62/PLOSOne_formatting_sample_title_authors_affiliations.pdf>

**Response: We have checked the PLOS ONE’s style requirements and made the corrections.**

2. Please provide additional details regarding participant consent. In the ethics statement in the Methods and online submission information, please ensure that you have specified (1) whether consent was informed and (2) what type you obtained (for instance, written or verbal, and if verbal, how it was documented and witnessed). If your study included minors, state whether you obtained consent from parents or guardians. If the need for consent was waived by the ethics committee, please include this information.

If you are reporting a retrospective study of medical records or archived samples, please ensure that you have discussed whether all data were fully anonymized before you accessed them and/or whether the IRB or ethics committee waived the requirement for informed consent. If patients provided informed written consent to have data from their medical records used in research, please include this information.

**Response: Our study includes aggregated data (not individual level data) publicly available from GBD website, and ethics statement do not apply. Because of this reason we have selected “N/A” in the online submission form.**

3. In your Data Availability statement, you have not specified where the minimal data set underlying the results described in your manuscript can be found. PLOS defines a study's minimal data set as the underlying data used to reach the conclusions drawn in the manuscript and any additional data required to replicate the reported study findings in their entirety. All PLOS journals require that the minimal data set be made fully available. For more information about our data policy, please see <http://journals.plos.org/plosone/s/data-availability>.

Upon re-submitting your revised manuscript, please upload your study’s minimal underlying data set as either Supporting Information files or to a stable, public repository and include the relevant URLs, DOIs, or accession numbers within your revised cover letter. For a list of acceptable repositories, please see <http://journals.plos.org/plosone/s/data-availability#loc-recommended-repositories>. Any potentially identifying patient information must be fully anonymized.

**Response: Our study included use of aggregated secondary data already publicly available from GBD website.**

**Reviewer #1**

**MAJOR COMMENTS**

1. The authors have not stated the sampled populations, inclusion criteria, and sampling strategy to select the desired number of samples from the GBD database. Therefore, sampling strategy and exclusion criteria are fundamentals of sampling strategy and need to be stated in the manuscript transparently and using complete and clear descriptions.

- **Response: Thank you for this comment. This is a secondary data analysis using Global Burden of Disease database. To add clarity to the readers we have now divided the methods section into two subsections- ‘data source’, and ‘analysis’, and have included additional details.**

2. Why did the author(s) include all ages instead of using a certain target age group? That might have reflected the heterogeneity and large variation of data. In addition, physiologically there is more likely to be obese with an increase in age, whilst the undernutrition problem is more prevalent in under 5 children. Therefore, the findings of this study could be affected by this phenomenon.

- **Response: We used age standardized rates for overall trend analysis. We understand age-wise variation is important in case of malnutrition and high BMI, and have presented age wise distribution of DALY for both in the figure 2.**

3. Overall, the method section needs to revise extensively. The methods section needs to be reported in a way that another research with access to the same database would be able to reproduce the (sub) sample used in this study. For instances, data extracting procedure, own sampling strategy for secondary data, description of study variables, and statistical analyses used for this study, etc.

- **Response: Based on your comment #1, and this comment, we have revised the structure of our methods section. We have provided the link to the website where the data was downloaded from, specified the indicators, year, country, and measures (rate per 100,000) which ensures reproducibility of our analysis.**

4. Since this study is not based on the multi-country analysis, why did the author(s) include the other south Asian country's data in the result? This is not in line with the study objectives.

- **Response: We included results from other South Asian countries to compare where Nepal stands. We mentioned this in line 83 in the introduction section, and in line 104 in the methods section.**

5. The entire paper could benefit from additional proofreading and grammatical correction to improve clarity and directness.

- **Response: Thank you for your comment. We have extensively copy edited the manuscript this time around.**

**MINOR COMMENTS**

**Abstract**

6. Overall abstract section needs to be reformatted like a background, methods, results, and conclusions.

- **Response: Thank you for this helpful note. We have now added those subsections in the abstract.**

7. Replace the word “report” with “identify or examine”

- **Response: We have edited it in the new version.**

8. Description of results in the abstract is mostly subjective. It is recommended to include objective measures of the results in this section.

- **Response: We have added numbers in the results section in the current revised manuscript.**

9. I would recommended to write clear and concise conclusions with brief recommendation.

- **Response: Thank you. We have edited our conclusion section.**

**Background**

The authors start from the macro idea, contextualize, and even identify the problem. The section is finished with the justification and the main objective of the work, there are some concerns that have to be addressed.

10. The authors should provide the tangible rationale of the study. Why does this study need to conduct? What is already known? What do new findings implicit? In the background, all these things need to be well addressed.

- **Response: We revisited the introduction section and made minor changes. Overall we felt it does answer questions raised by the reviewer.**

11. In the sentence “This double burden of malnutrition has been observed in many developing countries including countries in South Asia, including Nepal [6–9]” of these citations, number 8 study is not conducted in South Asia. Please revise it.

- **Response: We have revised the above mentioned reference.**

**Results**

12. In figure 2, along with the age-wise distribution of PEM and BMI, it would be better to present gender-wise too.

- **Response: We have presented gender wise differences in the trend in PEM and high BMI in figure 1 already.**

13. Only subjective results are presented for table 3, it is recommended to write numbers too.

- **Response: We do not have a Table 3, but we assumed you meant figure 3. Based on your feedback we added values for each bar in the graph.**

**Discussion**

14. The overall discussion needs to be specific. It could be more concise by only mentioning of most important discussion.

- **Response: We have extensively edited the discussion section in response to reviewers’ comments.**

**References**

15. Reformat all references with PLOS ONE referencing style and revise the references # 3, 9, 12, 14, 16,17,18, 20, 25, 32, 42 etc….

- **Response: Thankyou for addressing it. We have extensively revised the references section.**

—-------------------------------------------------------------------------------------------------------------------

**Reviewer #2:**

1. The basic guidelines of research paper submission need to be followed. There is no any line numbering. It would have been easier to give suggestion indicating the line number. Please check the submission guidelines

- **Response: We appreciate the note, and have added line numbers in the revised version of the manuscript.**

2. Title of the study and the content present in the paper do not match.

- **Response: Based on your feedback we have edited the title in this revised version.**

3. Methodology section is like nonexistent in the paper. There is no information about sample size, sampling strategy, inclusion and exclusion criteria of the study. There is no information on any predictor or outcome variable while discussion section is filled with various predictors of high BMI and PEM. How was the statistical analysis done? The questions like: How were the data extracted and what software was used for analysis, remain unanswered. Methodology need to be explained well enough to make the paper more transparent and meet the criteria of replicablility.

- **Response: Thank you for this comment. This is a secondary data analysis using Global Burden of Disease database. To add clarity to the readers we have now divided the methods section into two subsections- ‘data source’, and ‘analysis’, and have included additional details. In this revised version we have now provided the link of website where the data was downloaded from. We have specified the indicators, year, country, and measures (rate per 100,000) which ensures reproducibility of our analysis**.

4. BMI and PEM are quantitative variable. They require objective measurement and still they are prone to various errors. BMI and PEM are age sensitive. It would be interesting if the researcher had explained specifically on how information on PEM and BMI were assessed in GBD studies. Also, please provide clear operational definition of PEM and BMI.

- **Response: We agree that BMI and PEM are age sensitive, and thank you for pointing that out. We have now edited the methods section and have specified methods for age groups (>=20 years, and <=19years) for BMI.**

**About operational definition, unfortunately because it is a secondary data analysis using GBD data, we could only use the definition provided by the GBD.**

**In lines 80-82 we have clarified that we used PEM and high BMI as proxy indicators for undernutrtion and overnutrition. *“We used Protein-Energy malnutrition, a primary form of malnutrition in Nepali children***[***[15]***](https://paperpile.com/c/pxCO0Q/4Col) ***as a measure of undernutrition, and high body mass index (BMI) as a measure of overnutrition.*”**

5. The discussion section in research paper is basically based on findings of the research study and then one can explain how they fit with existing research and theory. The discussion in this research study is completely out of context. The aim of the study is trend analysis while discussion section was all about factors associated with BMI and PEM. In the discussion section, defend the findings of your own study than explaining results of other studies. Target population in the study is too wide/ vague.

- **Response: We have extensively edited the discussion section in response to reviewers’ comments.**

6. The referencing style does not follow the guidelines given by PLOS ONE. For instance, reference number 18 is about a research article while in the reference section only URL is copy pasted. Please recheck your reference section.

- **Response: Thank you for raising this. We have edited the references section in the current revised manuscript. We hope that we satisfyingly addressed them.**
