# Supplementary material for: Double burden of malnutrition in Nepal: A trend analysis of protein-energy malnutrition and High Body Mass Index using the data from Global Burden of Disease 2010–2019
Source: PLoS One. 2022 Sep 29;17(9):e0273485. doi: 10.1371/journal.pone.0273485 (PMC9521909; doi:10.1371/journal.pone.0273485)
Supplement: Attachment — Submitted filename: Response.docx [file pone.0273485.s003.docx]

a) Are these third party data (i.e., data not owned or collected by the author(s)?

**Response: Yes these are third party data, extracted from IHME GBD database**

b) If these are indeed third party data, please explain how others can access the specific datasets related to your study. Please confirm that others would be able to access these data in the same manner as the authors. Please also confirm that the authors did not have any special access privileges that others would not have.

**Response: The Global Burden of Disease data are freely available for everyone to download from the GBD results tool (**[**https://ghdx.healthdata.org/gbd-results-tool**](https://ghdx.healthdata.org/gbd-results-tool)**). User account is not needed. Researchers can select the required year, sex, age group, location, metrics, measures, causes, and risk factors of their interest. Once the selections have been made, the data can be downloaded in CSV file format by providing email address. When the file is ready for download the website sends an email with the link to download the data. The downloaded data can easily be opened in Excel for analysis or other statistical software.**
